# The evolution of theory of mind (ToM) within the evolution of cerebellar sequence detection in stone-tool making and language: implications for studies of higher-level cognitive functions in degenerative cerebellar atrophy

**DOI:** 10.1186/s40673-019-0101-x

**Published:** 2019-06-24

**Authors:** Larry Vandervert

**Affiliations:** American Nonlinear Systems, 1529 W. Courtland Avenue Spokane, Spokane, WA 99205-2608 USA

**Keywords:** Cerebellum, Cerebro-cerebellar blending, Cerebro-cerebellar decomposition, Degenerative cerebellar atrophy, Stone-tool evolution, Sub-vocal speech, Theory of mind (ToM), Verbal working memory

## Abstract

**Introduction:**

Within the context of Clausi, Olivito, Lupo, Siciliano, Bozzali and Leggio’s (Cell Neurosci 12:510, 2019) insightful study of how prediction of theory of mind (ToM) is compromised in degenerative cerebellar atrophy, this article describes how prediction can also be understood as the cerebro-cerebellar system’s capacity to rapidly shift attention to manipulate cause-and-effect relationships embedded in language.

**Method:**

The evolution of the capacity of ToM is described within the evolution of stone-tool making, language, and the origin of the phonological loop in verbal working memory. Specifically, it is argued that this evolutionary framework offers a way to get further inside the prediction process by illuminating how sub-vocal speech evolved during stone-tool evolution due to its adaptive refinement of early human ability to manipulate and hold in memory progressively more detailed cause-and-effect relationships in the origin of verbal working memory.

**Conclusion:**

The addition of sub-vocal speech/cause-and-effect relationship to the analysis of prediction provides an evolutionary model of the mechanisms of ToM, which, in turn, brings forward additional cerebro-cerebellar mechanisms which can (1) further support Clausi, Olivito, Lupo et al’s findings and (2) shed light on additional mechanisms that might further clarify what might be behind cerebellar dysfunction in the construction of ToM. Problems encountered by cerebellar degenerative atrophy patients with the Faux pas test and Advanced ToM task with unexpected events may stem from a combination of an inability (1) of their cerebellar internal models to rapidly switch attention among cause-and-effect elements of the stories and (2) to extend cerebellar internal models to the prediction of the resulting similar but unexpected events. That is, with both (1) and (2) occurring at the same time, alternative meanings of causes and effects might be missed in both automatic and consciously manipulated sub-vocal verbal working memory. A method to measure sub-vocal speech in this context is suggested.

## Introduction

Recently, Clausi, Olivito, Lupo, Siciliano, Bozzali and Leggio [1] published an insightful article on the role of the cerebellum in higher-level cognitive functions in patients with degenerative cerebellar atrophy. Within the framework of recent research on the “social cerebellum,” their research extends the cerebellum’s well established motor control roles to analogous/homologous in social thought and emotion. They do this by describing how cerebellum-driven prediction is involved in constructing Theory of Mind (ToM) (one’s simulative capacity to make inferences about the mental states of others), and by investigating how degenerative cerebellar atrophy influences the production of ToM. Specifically, they studied how cerebellar dysfunction might influence prediction and inferences in ToM by disrupting the learning and processing of cerebellar internal models.

At the higher levels of the construction of ToM, Clausi, Olivito, Lupo et al. [1] measured the capacity of degenerative cerebellar atrophy patients (CB’s) to interpret and make inferences about the thoughts, feelings and motives of others in stories comprising social situations. The stories were either (1) read to the subjects while subjects also read along and could check back on elements of the stories (the Faux pas test) or (2) simply read to subjects (the Advanced ToM task). To test the capacity of ToM construction, subjects then answered questions about whether they understood social inferences and attributions of story characters. In these high-level cognitive demand stories, CB’s showed diminished ToM construction ability. Clausi, Olivito, Lupo et al. concluded that in the presence of highest-level predictive load (as in *unexpected* story events in both the Faux pas test and the Advanced ToM task) cerebellar error signals are missed in social interpretation and inference.

## Purpose

This article offers a complementary approach to Clausi, Olivito Lupo et al. [1] but differs importantly in its approach to the nature of what, precisely, is predicted by cerebellar internal models that are formed in the development of the capacity for modeling the mental states of others in social ToM. This analysis is presented to help further clarify how prediction in cerebellar internal models might be compromised in cerebellar degenerative atrophy patients during the high level cognitive demands of the Faux pas test and the Advanced ToM task.

This article proposes to clarify this issue in three ways. *First*, it provides a framework for ToM that can offer clues to how the capacities for its “mind reading” functions were selected through cerebellar sequence detection during the evolution of stone-tool making and language. This evolution of stone-tool making and language will be described within the framework of the highly documented evolutionary neuroscience of cumulative culture proposed by Stout and Hecht [2]. Within this framework, the natural selection of mental and social elements of ToM construction can be identified within the learning of cerebellar internal models, their automaticity, and their connection with the prefrontal cortex [3, 4]. Cerebellar internal models were described by Ito [3, 4] as cerebellar representations of motor and mental models going on in the cerebral cortex. According to Ito, cerebellar internal models are learned in and mediated by cerebellar neural assemblages called microcomplexes.

*Second*, it provides a parallel natural selection of language and verbal working memory within stone-tool evolution which describes how the capacity for cerebellar internal models of verbal working memory (and complex, sub-vocal, silent inner speech [5]) originated and were progressively selected toward higher levels of social mentalizing [6, 7] and, thereby, toward higher level constructions of ToM. Following Vandervert [6] it will be shown how cerebellar internal models for repetitive, rapid shifts in attention required in stone-tool making may have been selected to accomplish higher levels of mentalizing. Vandervert proposed that this occurred through progressively more partitioned (decomposed) visual-spatial working memory and vocalizations necessary for likewise more detailed (micro-level) *cause-and-effect* manipulations required for rigorously repetitive, socially mediated stone-tool making. Accordingly, this adaptive, socially mediated evolution of working memory can be seen to have driven the evolution of the capacity for progressively more detailed constructions of ToM over eons of the evolution of stone-tool making perhaps beginning about 1.7 million years ago [8, 9]. It will be seen later in this article that this stone-tool/language co-evolutionary basis of ToM provides a way to further get inside the language-mediated cause-and-effect basis of subject’s capacities to simulate and interpret unexpected events in the stories presented in the Faux pas test and the Advanced ToM task.

*Third*, the idea that verbal working memory is the essential building block of the higher-levels of mentalizing in ToM can be helpful to the design of future studies of the cerebellum’s role in the construction of ToM. The analysis of the effects of the different phases of acquisition in verbal working memory and cerebellar internal models that drive silent speech (see, for example, [5]), especially those related to new or unexpected cause-and-effect relationships encountered by subjects in the Faux pas text and the Advanced ToM task, could greatly clarify these research efforts.

### The evolution of theory of mind (ToM) within the evolution of cerebellar sequence

#### Detection in stone-tool making and language

Stout and Hecht [2] described the overall *collaborative social demands* of stone-tool knapping that are required for the learner to achieve a high-level of competence. They took care to accurately describe this situation in the following two paragraphs; their description is repeated in detail here so that it can be closely tied into cerebro-cerebellar learning below:

Knapping is a “reductive” technology involving the sequential detachment of flakes from a stone core using *precise ballistic strikes* [italics added] with a handheld hammer (typically stone, bone, or antler) to initiate *controlled and predictable* [italics added] fracture. This means that small errors in strike execution can have catastrophic, unreversible effects. Experiments by Bril and colleagues have shown that fracture prediction and control is a demanding perceptual-motor skill reliably expressed only in expert knappers [10, 11]. Building on this work, Stout and colleagues [12–14] found that even 22 mo (x̄= 167 h) of knapping training produced relatively little evidence of perceptual-motor improvement, in contrast to clear gains in conceptual understanding.

The key bottleneck in the social reproduction of knapping is thus the *extended practice* [italics added] required to achieve perceptual-motor competence. This requires mastery of relationships, for example between the force and location of the strike and the morphology, positioning, and support of the core [11, 15, 16], that are not perceptually available to naïve observers and cannot be directly communicated as semantic knowledge. Attempts to implement semantic knowledge of knapping strategies before perceptual motor skill development are ineffective at best [17, 18], and such knowledge decays rapidly along knapping transmission chains when practice time is limited, even if explicit verbal teaching is allowed [19]. For *observational learning* [italics added], the challenge is to translate visual and auditory information of another’s actions to appropriate motor commands for one’s own body. This may be accomplished by linking the observed behavior with preexisting internal models (Stout and Hecht are referring to models in the cerebral cortex here, but as will be described below this point applies equally to cerebellar internal models] of one’s own body and actions through associative learning and stimulus generalization [20, 21]) …. These learning challenges call for an interactive approach that alternates social-learning opportunities (observation, instruction) with motivated individual practice [22], as commonly seen in coaching and apprenticeship practice. (p. 7862–7863).

In sum here, the social reproduction of knapping skill requires extended practice involving the highly detailed “sequential detachment of flakes from a stone core using precise ballistic strikes.”

Akshoomoff, Courchesne and Townsend’s [23] studied the cerebellum’s prominent role in the optimization of forward attentional control sequences in a broad variety of motor and cognitive processes, including both in cerebellar damage, and in normal working memory and language. Akshoomoff et al. described the learning of these sequences (sequence detection) in the following manner:

We hypothesized that the cerebellum does this by *encoding (“learning”) temporally ordered sequences* [italics added] of multi-dimensional information about external and internal events (effector, sensory, affective, mental, autonomic), and, as similar sequences of external and internal events unfold, they elicit a readout of the full sequence in advance of the real-time events [this readout is a *prediction*]. This readout is sent to and alters, in advance, the state of each motor, sensory, autonomic, attentional, memory, or affective system which, according to the previous “learning” of this sequence, will soon be actively involved in the current real-time events. (pp. 592–593).

It is clear from Akshoomoff, Courchesne and Townsend’s [23] findings and hypothesis that the required extended practice in learning the highly detailed sequential detachment of flakes in stone-tool making would heavily involve the cerebellum’s *encoding* and *prediction* of the fine social details related to the learner‘s detailed selective attention to the teacher’s knapping strikes (causes) and their effects. It should be noted that stating cerebellar encoding in terms of cause-and-effect relationships is critically important here because it links the progressive evolutionary selection of cerebellar sequence detection to Stout and Hecht’s [2] demonstrable stone-tool making events of that adaptive selection. Technically speaking, the fact that the “readout” of the learned sequence alters in advance (anticipates) system input for real-time events means the cerebellar encoding of sequences has indeed linked causes with effects for those events. This allows us to simultaneously see the meaning of sequence detection in stone-tool making, thinking, ToM, and so forth on one hand, and in the computations of the cerebellum on the other.

Vandervert [6, 7] proposed that these attentional control functions of the cerebellum drive (and drove the evolution of) the cerebellar forward internal model control of social behavior that occurs in the above-described social reproduction of knapping. This view comports completely with Van Overwalle, Manto, Leggio and Delgado-Garcia’s [24] hypothesis that the cerebellum operates as a “forward controller” in social interaction and self-action sequences. Specifically, they hypothesized that through this cerebellar forward control, the cerebellum predicts and anticipates in advance (sends to and alters in advance motor, emotional and cognitive systems) how both one’s self and others might behave and mentalize in ongoing situations. Their hypothesis further suggests that this cerebellar forward control “allows people to anticipate, predict and understand actions by the self or other persons and their consequences for the self, [and] to automatize these inferences for intuitive and rapid execution …”: (p. 35). This cerebellar forward control is precisely what is required for the learner to work through Stout and Hecht’s [2] earlier quoted “key bottleneck in the social reproduction of knapping” and achieve a high level of skill in stone-tool making.

The learning of stone-tool making is a rigorous, repetitive task which pushes the capacities of the learner’s rapid focusing and shifting of *attention* back and forth between the teacher’s knapping movements (causes) and their effects on the stone core on the one hand, and the learner’s own movements and their effects on the other. In the next section it will be proposed that the rapidity and complexity of this attention switching selected cerebellar decomposition and blending of existing sub-vocalization toward language evolution. Thus, within this framework of socially mediated cerebellar sequence detection, it is suggested that ToM evolved most significantly within the co-evolution of stone-tool making and language.

### Micro-level attention required in stone-tool knapping led to the high-level of ToM construction in the sub-vocal precursor of verbal working memory

In learning stone-tool making, micro-level, *precise ballistic strikes* of the teacher and their effects must be imitated. In this regard, the eye/hand/arm movements of the teacher may be understood as cerebellar *controlled objects* in the learner, through which the learner learns.^1^ In this regard, Wolpert, Doya and Kawato [25] proposed that a high level of “control” and observational learning related to the nonverbal behavior and intentions of others can be based on cerebellar internal models of one’s own motor system:

We hypothesize that … during action observation the motor system [one’s own motor system] can be used to understand the actions of others. This could be an efficient process because our CNS has learned to predict the *consequences of actions* [italics added] on our own body [as a collection of controlled objects] and this can be used to make accurate predictions about others. (p. 597)

Thus, in accordance with Akshoomoff, Courchesne and Townsend [10], Leggio and Molinari [29], and Wolpert, Doya and Kawato, [25], the collection of cerebellar internal models produced in the learner by observing the rapid shifting and focusing of attention back and forth between these two elements (cause-and-effect) is based on the learner’s own cause-and-effect system. Put simply, this shifting of attention between causes and effects produces the prediction, simulation, and inferences about the “mind” of the teacher (ToM) that is being internalized, that is, forming internal models of what Van Overwalle, Manto, Leggio and Delgado-Garcia’s [24] refer to as one’s autobiographical self.

The Evolutionary Effect of Rapid Shifts of Attention on Existing Visual-Spatial Working Memory and Vocalization.

Vandervert [6] argued that the detailed cause-and-effect relationships required in the attention-driven cerebellar modeling of stone-tool making led to the selective decomposition and blending of internal models [30–33] of early humans’ visual-spatial working memory and vocalization. This state of working memory likely existed in early humans approximately 1.7 million year ago with early intentional stone modification [34]. Vandervert [6] suggested that this early stone era was the basis of the earliest adaptive selection (decomposition and blending) toward sub-vocal speech. Through this decomposition and blending, cerebellar internal models for sub-vocal speech would have adaptively increased the detailed quality of prediction of the effects of stone manipulation. In addition, sub-vocal speech rehearsal during stone work would have helped retain simple cause-and-effect relationships in memory [5], and would have permitted mental manipulation, and autobiographical recording of those cause-and-effect relationships.

This overall evolutionary scenario is strongly supported by Baddeley, Gathercole and Papagno’s [35] proposal that “the primary purpose for which the phonological loop evolved is to store unfamiliar sound patterns while more permanent memory records are being constructed” (abstract).^2^ Baddeley, Gathercole and Papagno admittedly could not further articulate the mechanisms involved in the phonological loop. However, following the findings of Castellazzi, Bruno, Toosy, Casiragi, Palesi, Savini et al. [39], it is reasonable to suggest that new, repetitious words would be error-corrected and modeled in the cerebellum in relation to existing working memory. This scenario provides a direct neurological parallel to Baddeley, Gathercole and Papagno’s description of the purpose and operation of the phonological loop for acquisition of new word forms, a scenario that within Vandervert’s [6, 7] proposals places the evolutionary origin of the phonological loop as a concomitant to the rapid, complex attention shifting required in the evolution of stone-tool making.

The foregoing evolutionary scenario is also supported by Hecht, Murphy, Gutman et al.’s, [8] study of the human origins of object-directed grasping:

When copying others’ behavior, humans have a greater propensity for copying action details (*imitating*), whereas chimpanzees have a greater propensity for copying action outcomes (e*mulating*) [40]. Similarly, when monitoring their own behavior, humans have a bias toward monitoring kinematics, whereas chimpanzees have a bias toward monitoring goals [41]. Humans’ increased *attention* [italics added] to their own and others’ action details has been identified as a key factor in the emergence of imitation, cumulative culture, and the complex object-related behaviors they enable [42, 43] …. (pp. 14131–14,132).

It is proposed that these several lines of research can suggest that pre-language sub-vocal speech utterances first developed predominantly *not* for direct communication with others but, rather, for adaptive self-talk within the earliest stone-tool making. Nonetheless, one’s cerebro-cerebellar internal models for this repetitive self-talk would, following Wolpert, Doya and Kawato [24], have been used to model (infer, simulate) such task-specific self-talk in others, the earliest evolution of working memory-mediated ToM.

### A verbal working memory-phonological loop explanation for diminished ToM simulation ability among degenerative cerebellar atrophy patients (CB’s)

Within the foregoing stone-tool, sub-vocal speech-driven evolutionary scenario of the origin of ToM, it is suggested that in the high-load tasks of the Faux pas test and Advanced ToM task subjects must quickly learn and respond to nuances of *new* (unexpected) verbal accounts where the meaning (or direction) of cause-and-effect relationships are subtly switched. In this new, unexpected verbal situation, subjects must quickly switch attention and test the logic of alternatives associated with high-level cause-and-effect verbal information. For example, in the subjects’ own verbal working memories they must switch attention between “decision-making” processes ([44], p., 298) related to cause-and-effects going on among the story characters. Thus, subjects must (1) rely on flexibility in automaticity in cerebellar dynamics model memory [3, 4, 45] of representations in real-time, continuous and new verbal learning in working memory (self-talk) (a la Castellazzi, Bruno, Toosy, Casiragi, Palesi, Savini et al., [39], and, simultaneously (2) rehearse that new verbal account of the situation in silent sub-vocal speech in the cerebellum [5, 38] while continuing to listen to the story, and testing alternative logics (ideally automatically) until they are asked to respond to questions about the story. Thus, the requirement to be flexible enough [3, 4, 45] for attention to focus on the nuances of meaning of shifting cause-and-effect relationships would dramatically tax the capacity the phonological loop in the subject’s verbal working memory. It is important to recall here that the sub-vocal rehearsal of *new* vocalization or word information from others, which is extremely important in Clausi, Olivito, Lupo et al’s [1] experimental method, was proposed by Baddeley, Gathercole and Papagno [35] to be the evolutionary basis of the phonological loop within verbal working memory. That is, the task of learning new vocalizations adaptively selected toward the rehearsal function of the phonological loop.

It is proposed that both (1) the necessity of error-correction to automatic patterns of ToM simulation and (2) verbal rehearsal alternatives are at play to varying degrees depending on learning histories of subjects as determined by responsibility predictors based, in turn, on histories of error-correction as described by Wolpert, Doya and Kawato [25] in the HMOSAIC architecture. To determine which of these is the case for particular subjects, it is suggested that verbal rehearsal capacity among cerebellar degenerative atrophy subjects be studied in the Faux pas test and the Advanced ToM task. This could be accomplished using Marvel and Desmond’s [5, 38] theoretical perspective which supports the evolution of working memory as proposed by Baddeley, Gathercole and Papagno [35] and Vandervert [6, 7]. Marvel & Desmond proposed that:

The cerebellum enhances working memory by supporting inner speech mechanisms. This capability emerged from overt speech and motor systems as an evolutionarily adaptive way to boost cognitive processes that rely on working memory, such as language acquisition. ([38], p., 277)

Specifically, it is suggested that while Clausi, Olivito, Lupo et al. [1] provided important insights into the diminished performance of degenerative atrophy subjects, the stone-tool making origins of detailed cause-and-effect relationships and thereby language (the medium of ToM testing in Clausi, Olivito, Lupo et al.) strongly suggests that more on this diminished performance could be revealed in tests of working memory. To this end, it is suggested that this could be accomplished using Marvel and Desmond’s [5] methodology where subjects were tested on inner speech within verbal working memory. In part, they found that Broca’s area and the lateral superior cerebellum remained active during inner speech manipulation. Marvel and Desmond concluded that this activity “may present the ongoing creation of internal motor representations associated with inner speech—an effect that is augmented when information is manipulated. However, intense recruitment of this neural system can also signify one’s struggle to keep up with working memory demands” (p. 51). As described earlier in the introduction of this article, this is precisely the situation facing subjects in both tests in Clausi, Olivito, Lupo et al’s methodology.

## Conclusion

Evidence suggests that the evolution of highly rigorous and repetitive imitative requirements of stone-tool making, particularly in the last 1.7 million years, led to the human capacity of theory of mind (ToM). This era of stone-tool evolution produced cerebellar internal models of progressively more detailed cause-and-effect relationships which, in turn, adaptively led to progressively more detailed cerebellar internal models of sub-vocalization and the origins of verbal working memory.

Increasingly detailed sub-vocalization provided a cognitive framework upon which to produce progressively finer simulations the decision-making processes taking place in the minds of others (ToM) [44]. The initial phase in the origins of sub-vocal speech, while socially mediated in stone-tool making, was likely *not* adaptively selected for social communication, but for the learning of automatic manipulation of detailed cause-and-effect thought and enhancement of supportive working memory. Nonetheless, one’s cerebro-cerebellar internal models for this repetitive sub-vocalization (self-talk) could, have been used to model (infer, simulate) such task-specific self-talk in others, and thereby in the earliest evolution of working memory-mediated ToM. This idea jibes well with both (1) Van Overwalle, Manto, Leggio and Delgado-Garcia’s [24] hypothesis that the cerebellum operates as a “forward controller” in social interaction and self-action sequences, and (2) Wolpert, Doya and Kawato’s [25], suggestion that cerebellar internal models based on one’s own motor system can be used to make predictions about the nonverbal behavior and intentions of others.

Finally, it is suggested that studies of the cerebellum’s role in ToM can be informed by the analysis of sub-vocal speech. Problems encountered by cerebellar degenerative atrophy patients with the Faux pas test and Advanced ToM task with unexpected events may stem from a combination of an inability (1) of their cerebellar internal models to rapidly switch attention among cause-and-effect elements of the stories and (2) to extend cerebellar internal models to the prediction of the resulting similar but unexpected events [25, 45, 46]. That is, with both (1) and (2) occurring at the same time, alternative meanings of causes and effects might be missed in both automatic and consciously manipulated sub-vocal verbal working memory. It is suggested that this could be accomplished using Marvel and Desmond’s [5] methodology where subjects were tested on inner speech within verbal working memory.

## Footnotes

1 A controlled object is any entity (e.g., arm, leg, eye) including, as Ito [3] proposed, any mental model controlled by the cerebro-cerebellar system (e.g., [3, 25–27]). In the case of humanoid robotics, robot appendages are controlled objects controlled by computers [28].

2 The proposal of Baddeley’s [36] model of verbal working memory for social processing does not in any way discount the importance of studies that indicate social working memory mentalizing that takes place beyond the Baddeley model, for example, Meyer and Liebermann [37]. Rather, it is the intention of this article to comment on research designs within the operational specificity of verbal working memory methods, for example, that of Marvel and Desmond [5, 38].
