# Publisher Correction to: Cerebellum & Ataxias, volume 6

**DOI:** 10.1186/s40673-019-0104-7

**Published:** 2019-07-26

**Authors:** Cerebellum & Ataxias

**Affiliations:** London, UK

**Publisher Correction to: Cerebellum Ataxias (2019) 6:1**

**https://doi.org/10.1186/s40673-019-0101-x**

An error occurred during the publication of an article in *Cerebellum & Ataxias*. Article 10.1186/s40673-019-0101-x was published in volume 6 with a duplicate citation number.

In this correction article the old and new citation metadata are published in Table 1.

**Table 1:** Overview of incorrect and correct citation metadata

DOI	Incorrect citation number	Correct citation number
10.1186/s40673-019-0101-x	1	6

The original article has been updated. The publisher apologizes for the inconvenience caused to our authors and readers.

